# Phenotypic and genotypic characterization of ticks and tick-borne pathogens from cattle in selected villages of Greater Letaba Municipality in Limpopo Province, South Africa

**DOI:** 10.1007/s00436-024-08311-0

**Published:** 2024-08-05

**Authors:** Katleho Sechaba Monakale, Rae Marvin Smith, Realeboga Masego Gaorekwe, Maphuti Betty Ledwaba, Dikeledi Petunia Malatji

**Affiliations:** Department of Agriculture and Animal Health, College of Agriculture and Environmental Science, University of South Africa, 28 Pioneer Avenue, Roodepoort, 1709 South Africa

**Keywords:** 16S rRNA, Cattle, DNA, Ticks, Tick-borne pathogens

## Abstract

Ticks are blood ectoparasites that feed on domestic, wild animals and humans. They spread a variety of infections such as protozoa, viruses, and bacteria. Moreover, cattle reared by smallholder farmers are susceptible to ticks and tick-borne pathogens. Therefore, accurate identification of ticks and detection of tick-borne pathogens is crucial. The main aim of this study was to identify and characterize ticks and tick-borne pathogens from selected villages in Greater Letaba Municipality, Limpopo Province, using morphological and molecular techniques. A total of 233 ticks were collected from cattle and identified morphologically using appropriate morphological keys. The following tick species were identified: *Amblyomma hebraeum*, *Hyalomma rufipes*, *Hyalomma truncatum*, *Rhipicephalus appendiculatus*, *Rhipicephalus (Boophilus) decoloratus*, *Rhipicephalus (Boophilus) microplus*, *Rhipicephalus evertsi evertsi*, and *Rhipicephalus sanguineus. Rhipicephalus* spp. was the most common species accounting to 73.8% of the identified ticks. The genomic DNA was extracted from the whole tick for tick identification and from midguts of the ticks for the detection of tick-borne pathogens, followed by amplification and sequencing. A total of 27 samples were positive for tick-borne pathogens: 23 samples tested positive for *Theileria* and four samples tested positive for *Ehrlichia*. *Anaplasma* and *Rickettsial OmpB* could not be detected from any of the samples. There was no obvious grouping of ticks and tick-borne pathogens on the bases of their locality. The findings of this study confirm previous reports that indicated that cattle reared by smallholder farmers harbor various ticks and tick-borne pathogens of veterinary, public health, and economic importance. Regular monitoring of tick infestations in villages around the study areas is recommended to avoid disease outbreaks.

## Introduction

Ticks are blood-feeding ectoparasites of animals and humans and are vectors of many pathogens that can cause severe infectious diseases in both livestock and humans (Jongejan and Uilenberg 2004). They are not classified as insects but as arthropods, as they are closely related to mites, spiders, and scorpions. Ticks belong to the phylum Arthropoda, class Arachnida, subclass Acari, order Parasitiformes, and suborder Ixodida (Abubakar et al. 2018). There are about 900 tick species that have been identified globally, with 701 species thought to belong to the Ixodidae or hard ticks, 200 thought to belong to the Argasidae or soft ticks, and only one species thought to belong to the family Nuttalliellidae (Jongejan and Uilenberg 2004; Guglielmone et al. 2006). In addition, there are about 138 species of ticks that have been identified in the Afrotropical region (Guglielmonee et al. 2014).

Ticks are fast-multiplying parasites, and their habitat is composed of different environments for survival. They can be adapted to the new environment for survival and can survive either on the physical environment or host (Walker 2003; Nyangiwe et al. 2023). Over the past 30 years, there has been a significant growth in the distribution and abundance of various tick species. Such distribution and vector abundance are driven by several factors, predominantly climate change, vegetation, and host diversity (Léger et al. 2013). Apart from these factors, cattle movements are compounding the expansion further (Nyangiwe et al. 2013; 2017).

Under favorable conditions, the extreme reproductive output of ticks can generate high levels of transmission when pathogens are present (Barradas et al. 2021). Ticks carry and transmit a variety of pathogens such as viruses, bacteria, and protozoa. They are second to mosquitoes in importance as pathogen vectors and greatly impact human and animal health. There is a range of tick-borne diseases in South Africa associated with ticks infesting cattle which include agents of babesiosis, theileriosis, anaplasmosis, rickettsioses, heartwater, and Q-fever (Guo et al. 2019a).

Morphological characterization of ticks has been utilized as the traditional method to identify phenotype of ticks (Balinandi et al. 2020). These conventional techniques (ecological morphology) for species identification are limited when taxa are morphologically very similar, when specimens are damaged and when in immature stages or engorged (Nava et al. 2009). A study by Wanjira (2015) indicated that out of 98 tick species studied, 18 of them could not be morphologically identified as some were damaged beyond identification and others had morphological changes after feeding. In addition, Anderson et al. (2004) stipulated that this technique is very difficult to execute and does not give expected results and may lead to misidentification. Molecular techniques proved to be more convenient and reliable in studying the vectors of parasitic diseases. These techniques offer apparent opportunities as they are used to easily distinguish related taxa in ticks (Rumer et al. 2011). Moreover, molecular techniques, mainly those that are based on DNA sequences analysis, offer an alternative approach for the characterization of species (Nava et al. 2009). The aim of the current study was to morphologically and genetically identify ticks and tick-borne pathogens from cattle reared in by smallholder farmers.

## Materials and methods

### Study site

**Fig. 1:**
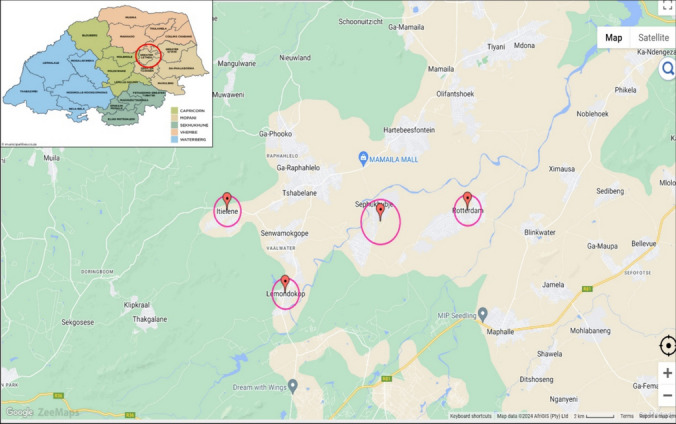
Map showing the study site within the Greater Letaba Municipality created using ZeeMaps

The study was conducted in selected villages of Great Letaba Municipality (Area, 1896 km^2^) of Limpopo Province, South Africa: Lemondokop (− 23.440813, 30.162331), Itieleng (− 23.404959, 30.110406), Rotterdam (− 23.396210, 30.291467), and Sephukubje (− 23.40461, 30.22073). The municipality is located within the North-Eastern quadrant of the Limpopo Province (Fig. 1).

### Sampling technique

Permission to conduct the study was granted from the University of South Africa’s Animal Research Ethics Committee (2022/CAES_AREC/191). The sample collection was performed according to SANS 10381 guidelines and regulations, and the methods of this study are reported according to ARRIVE guidelines. Cattle reared by smallholder famers were randomly selected from the cattle herds (not more than 4 animals per herd) at the local dip tanks using purposeful sampling which involved the selection of cattle that showed tick infestation. Ticks were collected in the winter season (July) during daytime from male and female cattle of different age-groups. They were pulled off manually from different body parts (genitals, anus, udder, hind quarter, front quarter) using dissecting forceps and then placed in sterile plastic vials and stored in 70% ethanol for retention until further use.

## Tick identification

### Morphological identification

Collected ticks were further kept at room temperature prior morphological identification. Ticks were then washed with sterile water to remove any residual debris such as animal skin or hair and rinsed once with 70% ethanol solution. All ticks were mounted on the slides and examined using stereoscopic dissecting microscope at the right magnification (× 40 and × 80). This was followed by identification of ticks by species using appropriate keys (Walker 2003).

### Molecular analyses of ticks

#### DNA isolation

The identified ticks were dissected, and their midguts were removed and used for tick-borne pathogen detection. The DNA was extracted from the rest of the tick tissue using Quick-DNA™ Miniprep Plus Kit (Zymo Research) following the manufacture’s protocol. The DNA concentrations were checked using Qubit 3.0 fluorometer (Life technologies), and DNA samples were stored at − 20 °C at Eureka laboratories (University of South Africa-Science campus) until further use.

#### Polymerase chain reaction (PCR) and gel electrophoresis

DNA samples were amplified using synthesized primers [forward: 5′-TTAAATTGCTGTRGTATT-3′] and [reverse: 5′-CCGGTCTGAACTCASAWC -3′] (Jizhou et al. 2014), and each tick species that was identified morphologically was represented by more than one sample. A total volume of 25 µl of the PCR mixture consisted of 2 µl of extracted genomic DNA, 1 µl of forward and reverse primers, 8.5 µl of distilled water, and 12.5 µl of PrimeSTAR Max DNA Polymerase (Takara). PCR amplification was conducted under the following conditions: initial step of 10 min at 95 °C, 35 cycles of denaturation at 95 °C for 30 s, annealing at 52 °C for 30 s and extension, and 1 cycle at 72 °C for 45 s. The reaction was completed by further extension of 7 min step at 72 °C and hold of 4 °C. A total of 5 µl of the PCR product was run on 1.5% of agarose (Condalab) dissolved in 100 ml of 1X TAE buffer, and 3 µl of gel stain (ethidium bromide) was added to the solution. Distilled water was used as a negative control, and 100 bp DNA ladder was used and run at 100 V for 60 min.

#### Sequencing and sequence analysis

PCR products that passed quality control (QC) test were sequenced using ABI3730XL genetic analyzer at Inqaba-Biotec, South Africa. A total of 64 (32 forward and 32 reverse) generated sequences were assembled and edited using the BioEdit version 7.2.5 (Hall 2013a). The consensus sequences were compared to published sequences obtained from National Centre for Biotechnology Information (NCBI), and multiple alignment was achieved using ClustalX2.1 program (Chisu et al. 2019). The maximum likelihood tree was constructed using Molecular Evolutionary Genetic Analysis (MEGA 11) software, and reference sequences were included using *Ixodes scapularis* (KT851344) as an outgroup. The default settings were used to draw the tree with 1000 bootstrap analysis and Kimura 2- parameter model (Kimura 1980) was used.

## Tick-borne pathogen detection

### DNA isolation

DNA was extracted from the tick midguts using Quick-DNA™ Miniprep Plus Kit (Zymo Research) following the manufacture’s protocol. DNA concentrations were checked using Qubit 3.0 fluorometer (Life technologies) and DNA samples were stored at − 20 °C until further analysis.

### PCR and gel electrophoresis

Tick-borne pathogens were detected and characterized by PCR using primers listed in (Table 1) which were developed previously (Omondi et al. 2017). To identify *Anaplasma* and *Ehrlichia*, the 16S rRNA gene was targeted (<200 bp) and rpmE-tRNAfMet intergenic spacer typing for Rickettsia which was developed previously was used. The apicomplexan hemoparasites, *Babesia* and *Theileria*, were amplified using previously described primers that are specific to the 18S ribosomal gene. For amplification, these are the thermal cycling conditions that were followed: initial denaturation for 15 min at 95 °C, followed by 10 cycles of 94 °C for 20 s, step-down annealing from 63.5 °C decreasing by 1 °C per cycle for 25 s for 11 steps, and primer extension for 30 s at 72 °C, followed by 25 cycles of denaturation at 94 °C for 25 s, annealing for 20 s at 50.5 °C, and extension for 30 s at 72 °C for 25 cycles, followed by a final extension at 72 °C for 7 min. Furthermore, a total of 5 µl of the PCR product were run on 1.5% of agarose (Lasec®) dissolved in 100 ml of 1X TAE buffer and 3 µl of gel stain (ethidium bromide) was added to the solution. Distilled water was used as a negative control, and 100bp DNA ladder (Biolabs®) was used.

**Table 1:** Primers used for detection of tick-borne pathogens

Pathogen/gene target	Primer pair	Amplicons size (bp)	Reference sequence
*Anaplasma* long 16S rRNA	Fwd: CGGTGGAGCATGTGGTTTAATTC Rev: CGRCGTTGCAACCTATTGTAGTC	330	KJ410254
*Ehrlichia* short 16S rRNA	Fwd:CGTAAAGGGCACGTAGGTGGACTA Rev: CACCTCAGTGTCAGTATCGAACCA	200	NR_074155
*Rickettsia/ompB*	Fwd:GTAAAATTACCGGTAAGGGTTATAGC Rev: ATACAAAGTGCTAATGCAACTGGG	200	CP001612
*Theileria/Babesia* 18S rRNA	Fwd:GAGGTAGTGACAAGAAATAACAATA Rev: TCTTCGATCCCCTAACTTTC	500	HQ684067

### Sequencing and sequence analysis

PCR products were sequenced using ABI3730XL genetic analyzer (Applied Biosystems) at Inqaba-Biotec, South Africa. Generated forward and reverse reads were assembled and edited using the BioEdit version 7.2.5 (Hall 2013a), and multiple alignment was achieved using ClustalX2.1 program (Chisu et al. 2019) before phylogenetic tree was constructed.

### Phylogenetic analysis

The generated DNA sequences were edited and compared with the sequences available in GenBank databases using the (NCBI) Basic Local Alignment Search Tool (BLAST) search engine (https://blast.ncbi.nlm.nih.gov/Blast.cgi), and multiple alignment was performed using the Geneious Prime 2023.2 (Biomatters inc.) with default parameters. The phylogenetic analysis was constructed using maximum likelihood tree based in MEGA 11, and the bootstrap values were estimated for 1000 replicates and Kimura 2-parameter model (Kimura 1980) was used.

## Results

### Morphological identification

**Fig. 2:**
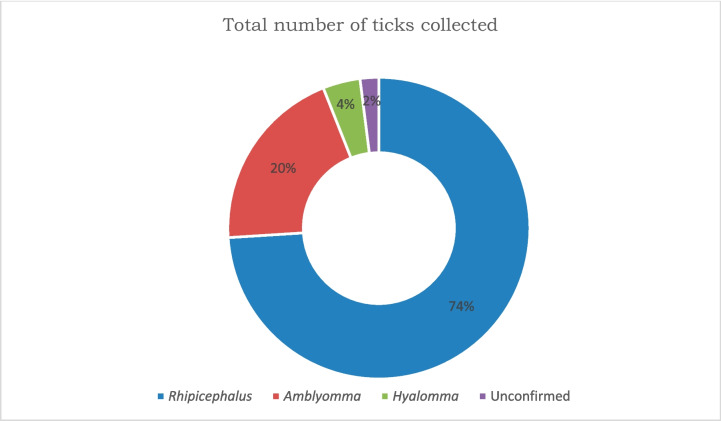
Total number of ticks identified per genera

A total of 233 ticks collected from four villages namely: Rotterdam, Itieleng, Lemondokop, and Sephukubje were morphologically identified. The identification of 62 ticks was confirmed by the Agricultural Research Council- OVR, South Africa. Figure 2 illustrates that the Ixodidae genera: *Amblyomma*, *Hylomma*, and *Rhipicephalus* were successfully identified using correct morphological keys (Walker 2003). *Rhipicephalus* was the most dominant with 73.8% of the total ticks belonging to this genus, followed by *Amblyomma* (20.2%) and *Hyalomma* (3.9%). Since some of the specimens were damaged and fully engorged, 2.1% of the ticks could not be identified. Cattle from Itieleng village consisted of 101 ticks identified, followed by Sephukubje (57), Rotterdam (50), and Lemondokop with 25 ticks identified.

**Fig. 3:**
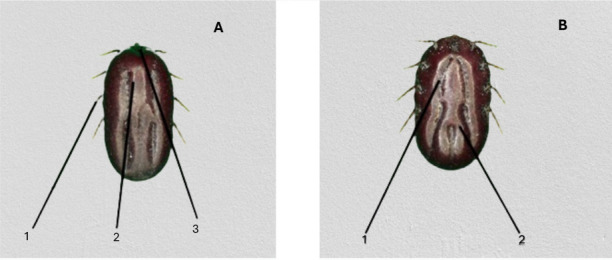
*Rhipicephalus (B) microplus* female. **A** Dorsal region: (1) the legs are short and more drawn back, (2) deep vertical scapula scapular grooves on the dorsal, and (3) visible small scutum. **B** Ventral region: (1) visible V-shaped grooves and (2) anus with a U-shaped groove

**Fig. 4:**
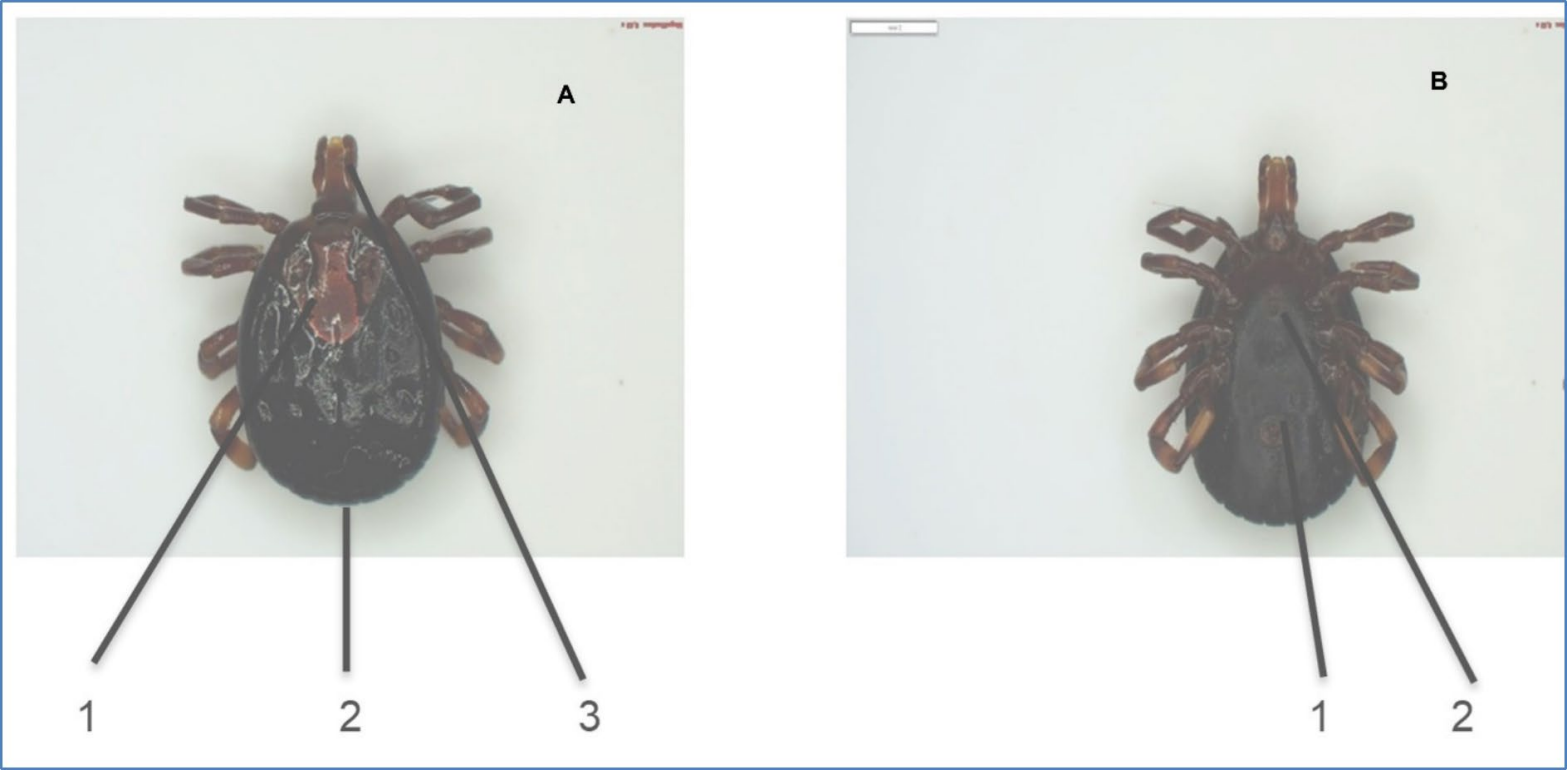
*Amblyomma hebraeum* female. **A** Dorsal region: (1) ornamented huge scutum, (2) long mouth parts, and (3) visible festoons. **B** Ventral region: (1) anal groove has a U shape and (2) genital aperture posterior lips have a narrow V shape

**Fig. 5:**
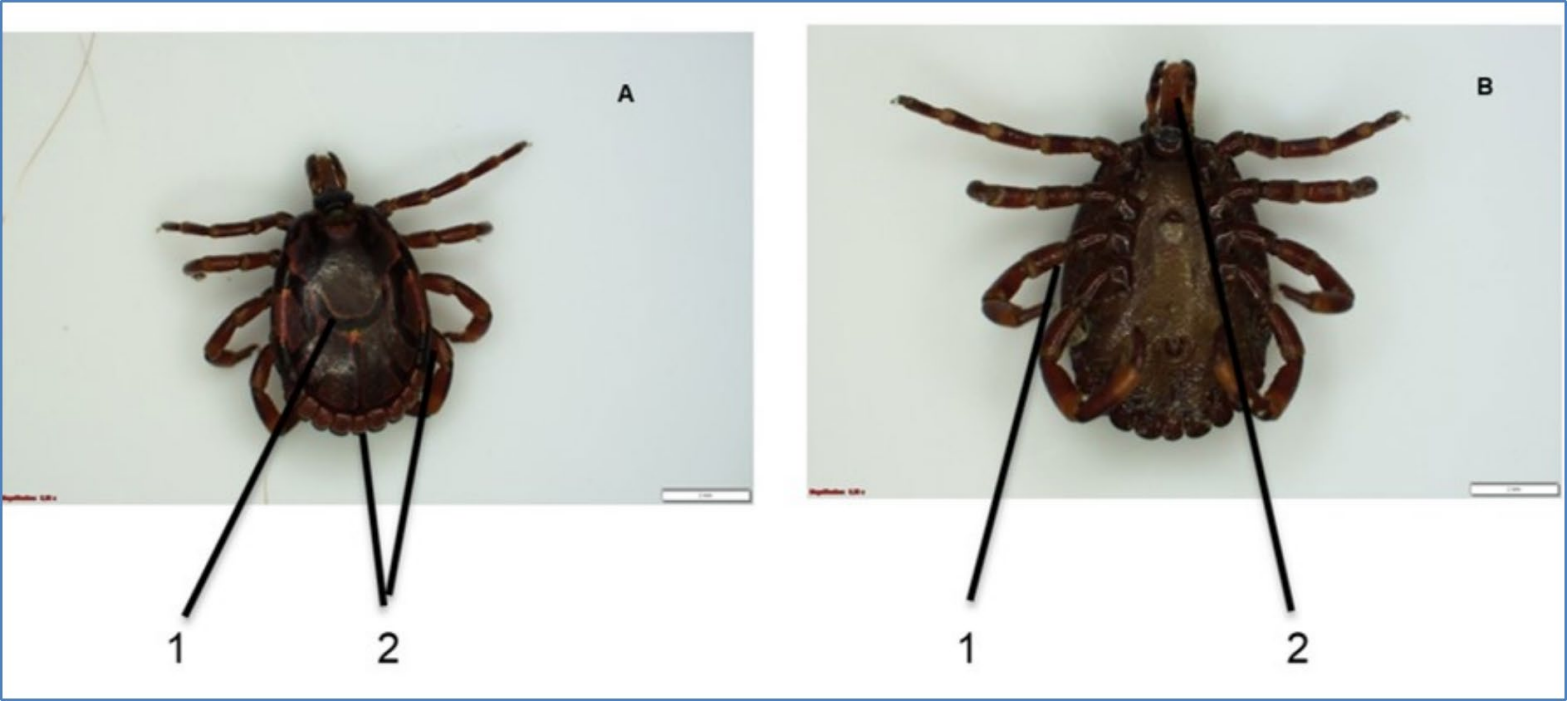
*Amblyomma hebraeum* male. **A** Dorsal region: (1) Conscutum is colored with large and complex colors, enameled and sparse distribution of punctation and (2) festoons are more visible (9 of 11 festoons are enameled, the two outermost festoons are not enameled). **B** Ventral region: (1) leg coloration is pale with rings and (2) the mouth parts are long and piercing sections are more visible

**Fig. 6:**
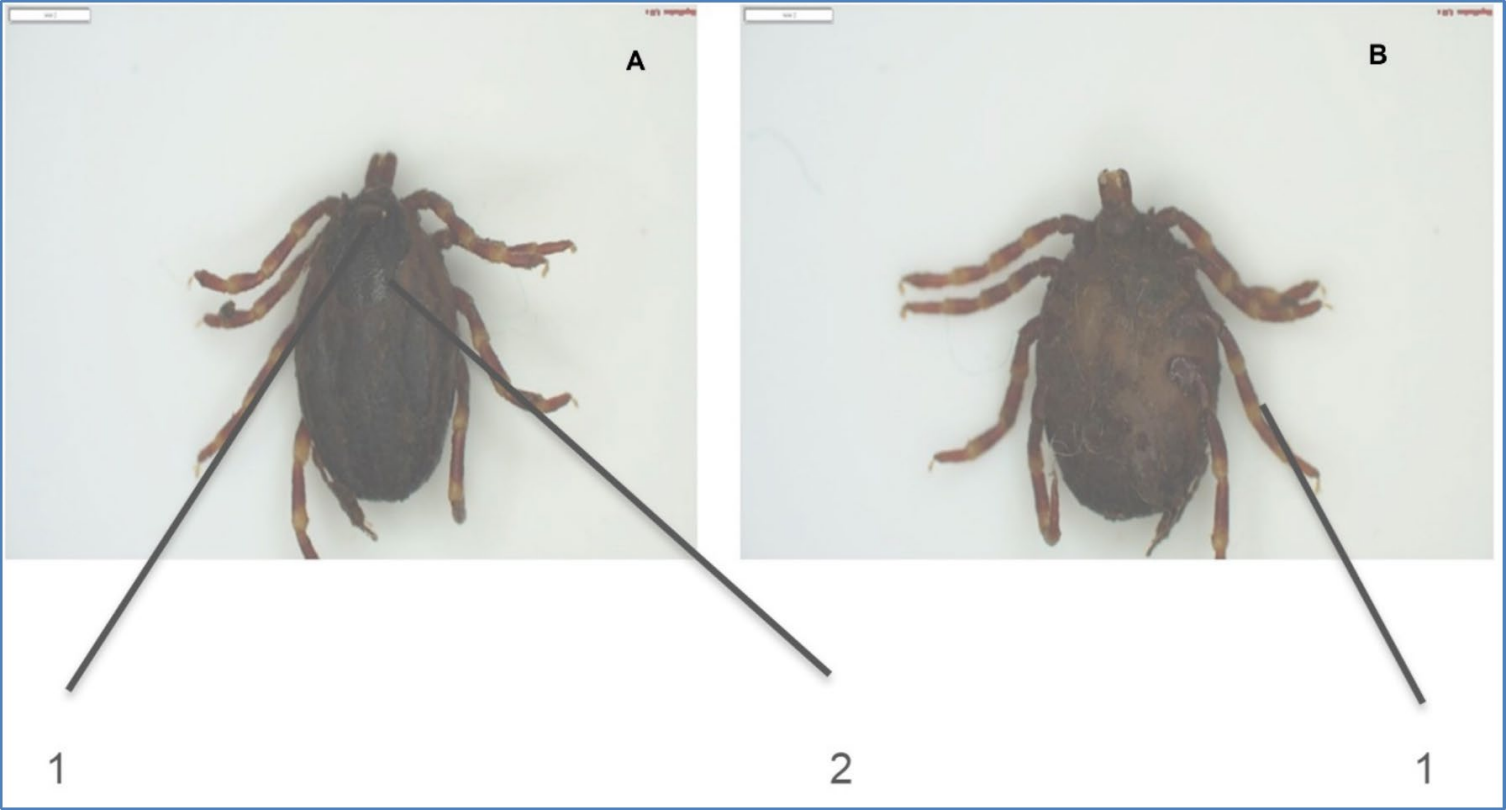
*Hyalomma rufipes* female. **A** Dorsal region: (1) scutum posterior margin is distinctly sinuous and (2) alloscutum is dark colored. **B** Ventral region: (1) leg colored with pale rings

**Fig. 7:**
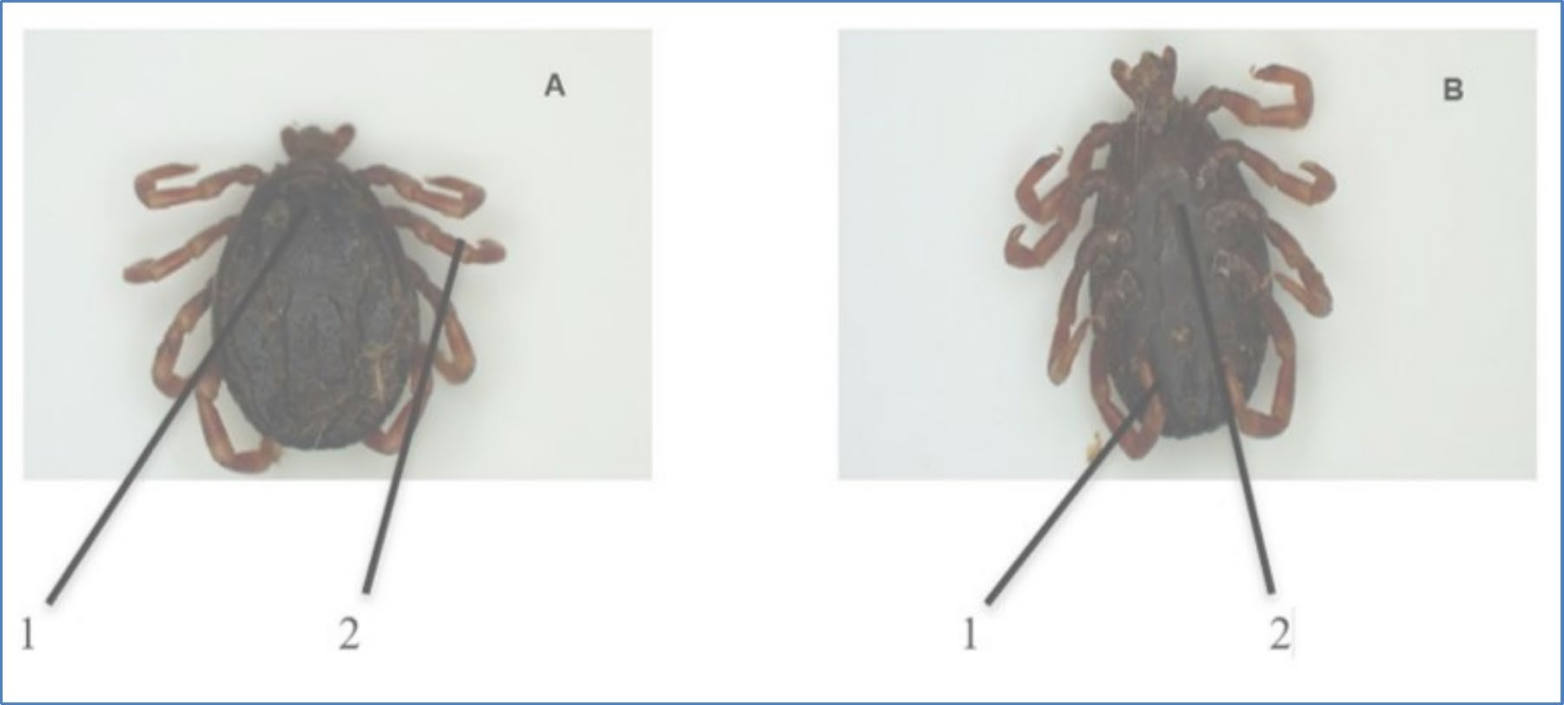
*Hyalomma truncatum* female. **A** Dorsal region: (1) Scutum is dark colored and posterior margin is distinctly sinuous and (2) legs are pale and long. **B** Ventral region: (1) adanal plates has square ends and (2) genital anterior groove is shallow

From the *Rhipicephalus* genus, the most prevalent species identified were *R. (B) microplus* (Fig. 3). The second prevalent species was *A. hebraeum* (Figs. 4 and 5), and the least prevalent was* H. rufipes* (Fig. 6) and *H. truncatum* (Fig. 7).

### Sequencing and phylogenetic analysis of ticks

**Fig. 8:**
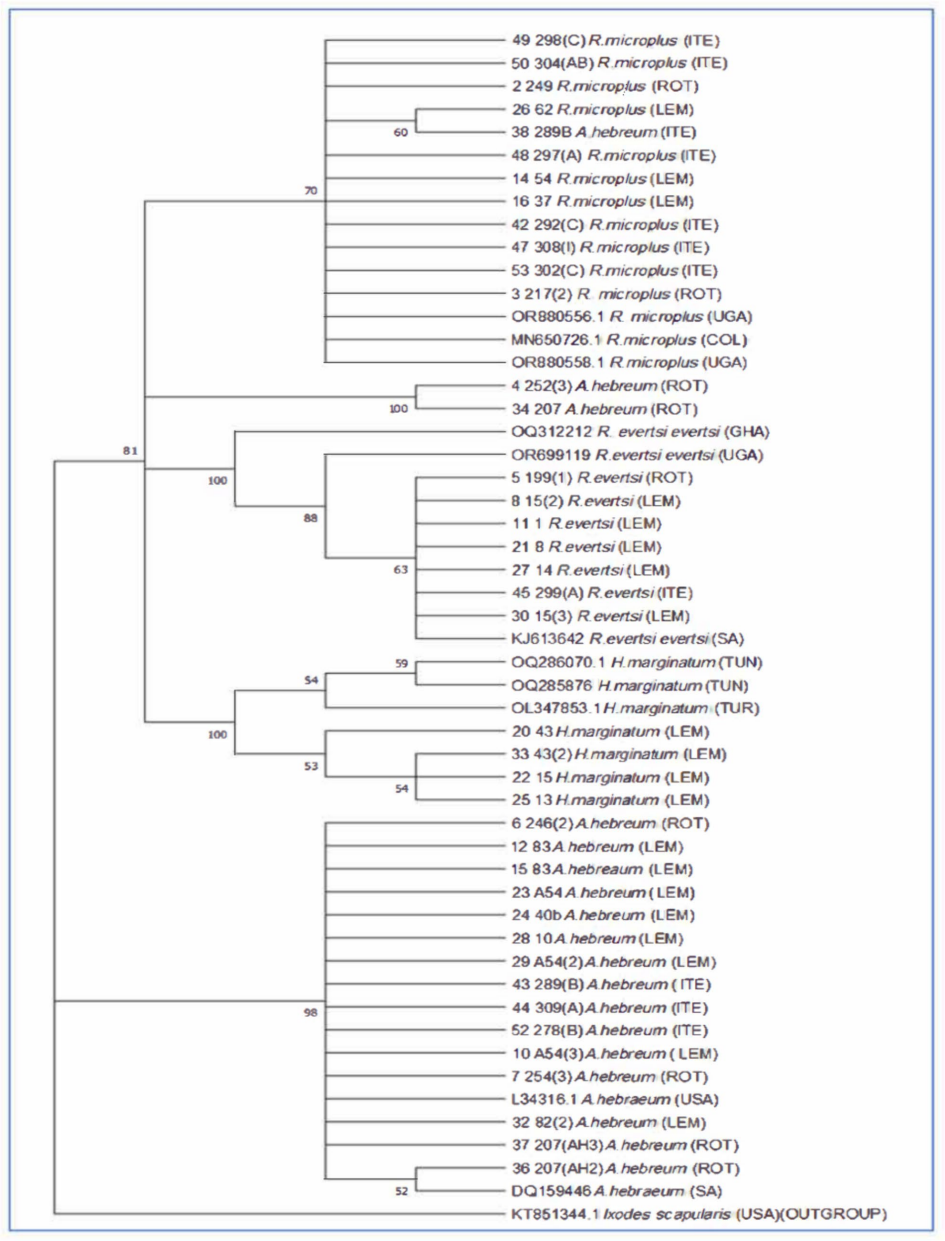
The phylogenetic tree of different tick species. The evolution history was inferred using the maximum likelihood. The percentage of replicates trees in which the associate taxa clustered together in the bootstrap test of 1000 replicates are shown next to notes. The tree was rooted with *Ixodes scapularis*

A total of eighty-six (86) forward (43) and reverse (43) sequences were generated. The phylogenetic relationship of the sequences was determined using the maximum likelihood tree using Mega 11. The reference sequences obtained from NCBI are listed in Table 2, and the genus *Ixodes* was used to root the tree (*Ixodes scapularis:* KT851344). *Rhipicephalus evertsi evertsi*, *R. (B) microplus*, and* A. hebraeum* clustered with reference sequences; however,* H. marginatum* ticks from the current study showed distinct clustering with sequences from NCBI (Fig. 8). There was no obvious grouping of ticks of the same species from the same villages, meaning that ticks from different villages grouped together. For example, *A. hebraeum* ticks from Lemondokop village did not form a distinct grouping/clade according to their locality, away from ticks collected from other villages. The same trend was observed with other tick species (*R. evertsi evertsi*, *H. marginatum*, and *R. (B) microplus*). It should be noted that two *A. hebraeum* tick samples from Rotterdam village formed their own clade away from other *A. hebraeum* tick samples from all villages.

**Table 2:** Published lineages of tick species used in the phylogenetic analysis and their accession numbers

Tick species	Accession number	Locations
*R. microplus*	OR880556	Uganda
*R. microplus*	MN650726	Colombia
*R. microplus*	OR880558	Uganda
*R. evertsi evertsi*	OQ312212	Ghana
*R. evertsi evertsi*	OR699119	Uganda
*R. evertsi evertsi*	KJ613642	South Africa
*H. marginatum*	OQ286070	Tunisia
*H. marginatum*	OQ85876	Tunisia
*H. marginatum*	OL347853	Türkiye
*A. hebraeum*	DQ159446	South Africa

### PCR amplification of tick-borne pathogens

The current study targeted only four tick-borne pathogens (*Anaplasma*, *Ehrlichia*, *Rickettsial OmpB*, and *Theileria*). A total of 27 samples showed to be positive for tick-borne pathogens: 23 samples tested positive for *Theileria* yielding amplicons of the expected size ~ 500 bp, and four samples tested positive for *Ehrlichia* at expected size of ~ 200 bp. *Anaplasma* and *Rickettsial OmpB* could not be detected from any of the sample, and all samples that did not amplify were excluded from further analysis.

### Sequencing and phylogenetic analysis (tick-borne pathogens)

**Fig. 9:**
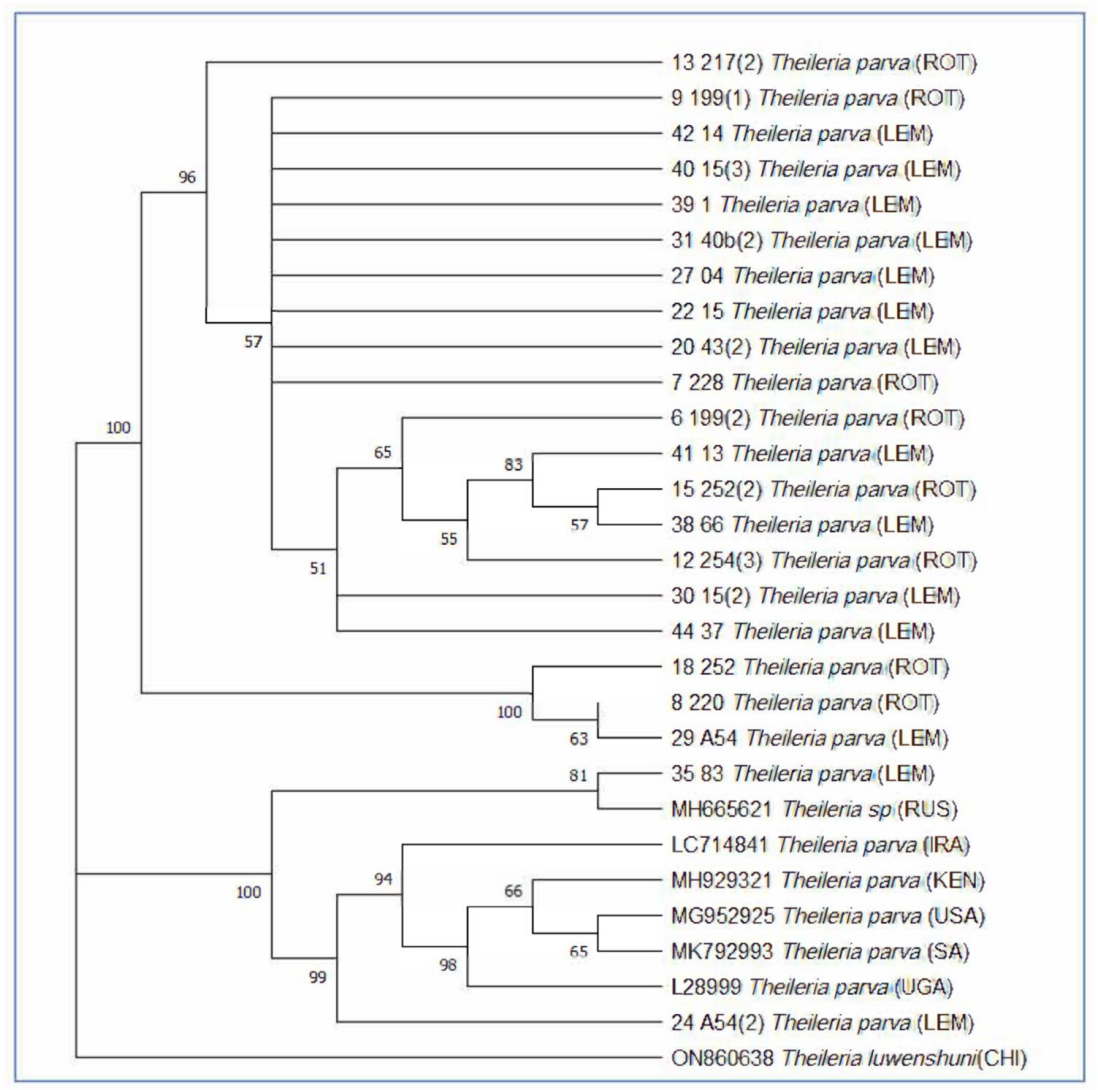
The phylogenetic tree of tick-borne pathogens was confirmed by maximum likelihood tree. The percentage of replicates trees in which the associate taxa clustered together in the bootstrap test of 1000 replicates are shown next to nodes

A total of 27 forward and reverse (27) generated sequence data were obtained from sequenced tick midguts for the detection of tick-borne pathogens. The consensus sequences were compared with the sequences from GenBank (Table 3) using *Theileria luwenshuni* (ON860638) as an outgroup. Only two samples from Lemondokop village clustered with the reference sequences (Fig. 9), and the rest of the samples did not share clades with published NCBI sequences. Notably, the reference sequence originating from South Africa (MK792993) did not cluster with the sequences from the current study. Similarly to the analyzed tick sequences, there was no obvious grouping of tick-borne pathogens on the bases of their locality.

**Table 3:** Published lineages of tick-borne pathogens used in the phylogenetic analysis and their accession numbers

Tick-borne pathogens	Accession number	Locations
*Theileria parva*	MH929321	Kenya
*Theileria luwenshuni*	ON860638	China
*Theileria parva*	LC714841	Iraq
*Theileria parva*	MG952925	United States of America
*Theileria parva*	MH665621	Russia
*Theilria parva*	L28999	Uganda
*Theileria parva*	MK792993	South Africa

## Discussion

Ticks are ectoparasites that mainly obtain their nutrition through blood sucking; these parasites are known to belong to two families, hard and soft ticks (Blisnick et al. 2017; Bhowmick and Han 2020). In this study, 233 ticks were identified microscopically, and 63 samples representing all the tick species were identified genetically. To the best of our knowledge, this is the first study conducted in the villages of Greater Letaba Municipality (Itieleng, Lemondokop, Rotterdam, and Sephukhubje) that detected and identified tick species and tick-borne pathogens. The current study identified ticks that belong to the genera *Hyalomma*, *Rhipicephalus*, and *Amblyomma*. These reported findings support the observations by Horak et al. (2018) that showed that ticks belonging to these genera are indigenous to South African ecology and affect wild and domestic animals.

The most prevalent tick species identified in the current study was *R. (B) microplus*, followed by *A. hebraeum*. This is in agreement with the studies reviewed by Makwarela et al. (2023) which indicated that in South Africa; the most prevalent tick species are *A. hebraeum*, *R. (B) decoloratus*, and *R. evertsi evertsi*. Similar results were also reported in Limpopo Province by Schroder and Reilly (2013) where they identified *R. (B) decoloratus* (67%), *A. hebraeum* (15%), and *R. evertsi evertsi* (8%). Furthermore, other tick species identified in the current study were found to be less prevalent in the sampled villages: *R. (B) decoloratus* (9%), *R. evertsi evertsi* (7%), *R. appendiculatus* (3.8%), *H. truncatum* (3%), *H. rufipes* (0.9%), and *R. sanguineus* (0.4%). In contrary, a study conducted by Halajian et al. (2016) reported that *R. appendiculatus* and *R. (B) decoloratus* were more prevalent in the province of Limpopo. These differences could be due to the type of vegetation animals graze on, which can hinder the adaptability of certain tick species or even the season sampling took place. A study conducted by Schroder and Reilly (2013) found that annual ecological seasons contribute to the prevalence of *A. hebraeum* ticks, which were identified more in wet and hot seasons. *Rhipicephalus appendiculatus* and *R. evertsi evertsi* were mostly prevalent in dry and cooler seasons, and *R. (B) decoloratus* was found to be adapted to all annual seasons. In the current study, sampling took place during the winter season, and it has been reported that the occurrence of ticks and tick-borne pathogens is higher in summer season (Atif et al. 2022). This was further supported by Okafor et al. (2018) who found that *A. marginale* was more prevalent in summer than other seasons. A study by Siddique et al. (2020) reported high incidence of ticks in summer, which was followed by autumn, spring, and winter season, respectively. This is in correspondence with the low prevalence of tick species observed in this study. Therefore, it is important to compare seasonal incidents of ticks in the studied villages of Greater Letaba Municipality to establish if they follow any trend.

Furthermore, Marufu et al. (2011) stated that tick adaptation can be influenced by type of pastures in the region, and the authors reported that prevalence *of R. (B) decoloratus*, *R. evertsi evertsi*, and *R. appendiculatus* was found to be adaptable to both sweet and sour rangeland while *Hyalomm*a species were found to be adapted only on sour rangeland. There is a need for future studies in the four villages to investigate the type of pasture in the area to establish the influence pasture has on tick prevalence in the area. In this study, *A. hebraeum* and *R. evertsi evertsi* were easily identified by their unique scutum and festoons described by Walker (2003). The author reported that male *A. hebraeum* and *R. evertsi evertsi* have unique characteristics that are useful for classification. The female *A. hebraeum* were easily identified with their huge ornamented scutum, long mouth parts, and visible festoons while female *R. evertsi evertsi* were easily identified with short mouth parts, visible dark scutum, and pale-orange legs (Horak·et al. 2014). Even though these features were helpful in identifying the above-mentioned tick species, these distinct features were difficult to identify on ticks that were damaged or engorged.

Notably, it was difficult to morphologically differentiate between *H. truncatum* and *H. rufipes* due to similar characteristics (leg colored with pale rings, short mouth parts, and sinuous scutum) that were present in these two species. Nava et al. (2009) indicated that identifying closely related tick species with distinct morphological features is a challenge even among experienced taxonomists. In addition, Abdi et al. (2011) attest to difficulties incurred in identification of *Hyalomma* spp. using nomenclatures. The above-mentioned authors also could not identify various ticks due to engorgement, physical damage, and some body parts that were missing. In this study, majority of *R. appendiculatus* ticks were engorged and could not be correctly identified under the microscope, and it was further observed that *R. (B) decoloratus* and *R. (B) microplus* had much similar features (short mouth parts, deep scapular grooves, and U-shaped genital aperture) which also contributed to difficulty in identifying these two species. Moreover, Abdi et al. (2011) noted that morphological changes can occur on the ventral and dorsal parts of ixodid ticks which may lead to misleading morphological identification.

In this study, ticks were first identified using morphological method before PCR amplification was conducted to confirm the finding by targeting 16S rRNA marker. Molecular analysis assisted with accurate identification of engorged, immature life stages, and damaged ticks. Several studies also support that morphological technique can be very difficult to execute and does not give expected results and may lead to misidentification (Anderson et al. 2004; Abdi et al. 2011; Couper and Swei 2018).

In the present study, partial sequence of 16S rRNA was used to identify tick species from different genera. This marker was also targeted by researchers in other studies (Muruthi et al. 2016; Omondi et al. 2017; Ringo et al. 2018) and have provided additional taxonomical knowledge and systematics of both ticks and tick-borne pathogens. Similarly to morphological identification, the marker assisted in the confirmation of tick species such as *A. hebraeum* and *R. (B) microplus*. These findings are similarly to those observed in a study by Pillay and Mukaratirwa (2020), which investigated ticks and tick-borne pathogens in Eastern Cape and found *A. hebraeum* to be the principal species in the region. Furthermore, *A. hebraeum* sequences generated from the current study showed homology ranging from 96 to 100% corresponding with NCBI reference sequences with these accession numbers: L34316, NC_067897, and KY457513. This suggests that the species could be classified in accordance with the genotypic identification provided in the constructed phylogenetic tree, which illustrated the species to be clustered accordingly in the classification group. These results agree with observations made by Balinandi et al. (2020) and Muhanguzi et al. (2020) who reported the clustering of ticks per species. However, one *A. hebraeum* spp. was observed to be clustering with *R. (B) microplus* and could be due to number of base-pairs blasted in GenBank. A study by Burger et al. (2012) also indicated that number of base-pairs could affect correct separation of species. *R. (B) microplus* is the second most identified tick species with three reported lineages (OR880556, OR880558, and OP909779) with similarity percentage of 100% and one lineage (MN650726) at 96.99% in correspondence with published sequences from GenBank. This corresponds with the findings of a recent study conducted in Limpopo Province which identified *R. (B) microplus* as one of the crucial ticks in the Limpopo Province (Frawley et al. 2024).

Tick-borne pathogens are a threat to various cattle breeds throughout the world, especially the cattle from villages due to the use of common pastures and drinking points that can enable infections among animals (Bell-Sakyi et al. 2004). In the current study, tick-borne pathogens were prevalent in two villages only: Lemondokop (59%) and Rotterdam (41%), and the most common tick-borne pathogen detected was *Theileria parva. Theileria parva* was also detected in cattle found in rural villages of KwaZulu-Natal (Yusufmia et al. 2010). These two studies support the finding by Uilenberg (1995), who reported theileriosis (caused by *Theileria parva*) as one of the pathogenic major tick-borne diseases found to affect livestock in South Africa. Other *Theileria* spp. (*Theileria taurotragi*, *Theileria mutans*, *Theileria velifera*) that are reported to cause non-pathogenic or mild theileriosis were not found in this study (Horak 1982), similarly to our findings. The tick-borne pathogen occurrence in cattle from the sampled villages could be ascribed to the detected tick vectors that are responsible for transmitting these parasites. For example, in South Africa, *Theileria parva* is caused by *R. appendiculatus* which was identified in this study. Cattle that recover from *Theileria parva* infection tend to be carriers of the pathogen for the rest of their lives. It is, however, not known if the cattle used in this study were carriers or not. Nonetheless, the phylogenetic analysis based on *Theileria parva* found that four clades were formed; however, they did not group following any pattern. Pathogens from different villages were clustered together which might suggest that the pathogens available in different villages are the same. *Ehrlichia* spp. was also detected in this study as one of the two economically important tick-borne pathogens. The occurrence of this pathogen was also reported by a study conducted by Mtshali et al. (2016) who identified *Anaplasma marginale*, *Theileria taurotrari*, and *Ehrlichia* spp. as the most common tick-borne pathogens identified in four provinces of South Africa: KwaZulu-Natal, Free state, Mpumalanga, and Eastern Cape.

Among the sampled households, each cattle had moderate to abundant number of ticks. The observed tick visibility can be related to poor tick control practices in the study areas. These practices can include among others uniformed spraying of acaricides, handpicking ticks, and lack of support from government veterinary services. Similarly, Nyangiwe (2017) in Eastern Cape found that village farmers lack knowledge or supplement government tick control by using alternative methods like used engine oil and jeyes fluid as control measures. However, Sichibalo et al. (2021) further reported that *Amblyomma* spp. and *Rhipicephalus* spp. showed tick resistance to registered acaricides in communal cattle production. In addition, Dzemo et al. (2023) reported that acaricides resistance occurs when cattle are dipped more than five times a year. The author further explained that repetitive use of acaricides with the same active ingredient increases the chances of developing acaricide resistance. Moreover, cross-border movement and communal grazing of cattle might also increase the chances of tick infestations in cattle (Byaruhanga et al. 2015). Furthermore, de La Fuente et al. (2015) indicated that vaccines with tick antigens are more cost effective and environmentally friendly with double effects as they reduce infestation and prevent ticks from transmitting tick-borne pathogens.

## Conclusion

The current study managed to shed some light on the incidents of ticks and tick-borne pathogens in Itieleng, Rotterdam, Sephukhubje, and Lemondokop villages. According to our knowledge, this research is the first to be conducted in the study areas and successfully explored a combination of molecular and morphology techniques to detect and identify ticks and their associated pathogens. The findings of this study will contribute additional information on the ecology of ticks and prevalence of tick-borne pathogens in the province of Limpopo. The data collected will assist in updating the host and geographic distribution records provided by previous studies in the province. Furthermore, this study forms basis for the implementation of effective control measures that can be used in the study areas. The four villages are serviced by the same local Department of Agriculture, Land Reform and Rural Development office, which will make it easy for the department to provide control intervention against the identified ticks as they appear to not be different between and within villages. It should also be noted that understanding relationship between ticks and tick-borne pathogens is crucial to evaluate possible transmissions and conducting informed strategies for treatments which could potentially reduce persistent infections and possible outbreaks of zoonotic diseases.

## Data Availability

The sequence data generated during the current study have been submitted to NCBI GenBank database under these accession numbers: PP999625—PP999628, PQ000332—PQ00035, PQ000885—PQ000924, PQ000980—PQ000983 and PQ001006—PQ001565.
